# Association between Aflatoxin M_1_ and Liver Disease in HBV/HCV Infected Persons in Ghana

**DOI:** 10.3390/ijerph13040377

**Published:** 2016-03-29

**Authors:** Clarrisa Afum, Lorene Cudjoe, Justin Hills, Raymond Hunt, Luz A. Padilla, Sarah Elmore, Abena Afriyie, Ohene Opare-Sem, Timothy Phillips, Pauline E. Jolly

**Affiliations:** 1 Department of Epidemiology, School of Public Health, Ryals Public Health Building, University of Alabama at Birmingham, 1665 University Boulevard Birmingham, Birmingham, AL 35294-002, USA; clarrisaafum@gmail.com (C.A.); lorenecudjoe@gmail.com (L.C.); jlhills14@gmail.com (J.H.); rbhunt@cchs.ua.edu (R.H.); apadilla@uab.edu (L.A.P.); aafriyie09@gmail.com (A.A.); 2 Veterinary Medicine & Biomedical Sciences, Texas A & M University, College Station, TX 77845, USA; SELmore@cvm.tamu.edu (S.E.); tphillips@cvm.tamu.edu (T.P.); 3 School of Medical Sciences, Kwame Nkrumah University of Science and Technology, Kumasi, Ghana; oparesem@hotmail.com

**Keywords:** liver disease, hepatitis B/C virus, aflatoxin M_1_, Ghana, hepatocellular carcinoma

## Abstract

Aflatoxins are produced by the fungi *Aspergillus flavus* and *Aspergillus parasiticus* and are common food contaminants in tropical developing countries. Extensive aflatoxin consumption has been shown to be highly associated with liver disease. A case-control study was conducted to determine the association between aflatoxin and liver disease in Kumasi, Ghana. A questionnaire was administered to examine socio-demographic characteristics and food storage and consumption practices, and urine samples were collected to measure levels of the aflatoxin metabolite (AFM_1_). Two hundred and seventy-six people participated in the study; 38 had liver disease (cases), 136 had neither hepatitis B/C nor liver disease (negative controls), and 102 were hepatitis B/C positive without liver cancer (positive controls). A much higher percent of participants in each group was male (76% of cases, 88% of negative controls and 65% of positive controls). Multivariate analysis showed that age was a significant predictor for being a case when cases were compared to negative controls. The odds of being a case was 70% less for participants aged 25–34 years (odds ratios (OR) 0.30; 95% confidence interval (CI) 0.10–0.88) compared to those ≥45 years. For cases; Akans were seven times more likely to have AFM_1_ levels below the median when compared to other ethnic groups (OR 7; CI 1.41–34.68). When cases were compared to positive controls, they were 2.29 times more likely to report awareness of aflatoxin contamination of groundnuts (95% CI 1.06–4.91). Cases were also two times more likely to report awareness of aflatoxin contamination of maize than all controls combined (95% CI 1.02–4.11). However, most cases reported that aflatoxin contamination does not cause sickness in humans. This shows that there is awareness of aflatoxin contamination without proper understanding of the serious potential adverse health impacts among these study participants. These findings indicate that educational interventions that stress the harmful health effects of aflatoxin in food, with an emphasis on the higher risk for males, are urgently needed. The reasons for lower aflatoxin levels among Akans need to be determined, and the findings used to design interventions that benefit other ethnic groups in the society.

## 1. Introduction

Aflatoxins are a group of extremely toxic metabolites produced by the common fungi *Aspergillus flavus* and *A. parasiticus*. Although the major aflatoxins (B_1_, B_2_, G_1_ and G_2_) occur together in various foods in different proportions, aflatoxin B_1_ (AFB_1_) is usually the predominant and most toxic form [1,2]. AFM_1_ is a major metabolite of AFB_1_ and is frequently excreted in milk and urine of humans, dairy cattle and other mammals that have consumed food or feed contaminated with aflatoxins [1]. The aflatoxin B_1_-8,9-epoxide, which is formed from aflatoxin B_1_ (AFB_1_) by cytochrome p450 oxidation, is highly reactive and can bind to DNA, RNA and proteins forming adducts and resulting in cancer and toxicity [3,4]. The conditions that favour growth of the fungi that produce aflatoxin are optimal in Ghana. Moreover, staple foods such as, maize, groundnuts and other grains are often contaminated with levels of aflatoxin that far exceed the 30 µg/Kg considered tolerable in food for human consumption by the FAO/WHO/UNICEF Protein Advisory Board [5]. The most severe effect of aflatoxin in the human body is seen in the liver, which is generally responsible for detoxifying chemical agents and poisons.

The adverse health effects of aflatoxins can be categorized as either acute or chronic. In acute aflatoxicosis, binding of the AFB_1_-epoxide to various cellular macromolecules leads to hepatocellular injury and death [6]. Acute aflatoxicosis manifests as severe, acute hepatotoxicity with a case-fatality rate of up to 39% [7,8]. Symptoms include acute liver damage, rapid progressive jaundice, edema of the limbs, high fever, abdominal pain, vomiting, fulminant hepatic failure and possibly death.

Chronic exposure to aflatoxin causes carcinoma of the liver [9], and both epidemiological and animal studies show that hepatitis B virus (HBV) and aflatoxin work synergistically to increase the risk of hepatocellular carcinoma (HCC) [10,11]. HCC is the fifth most frequently occurring cancer in the world [12], with an estimated 0.5–1 million cases annually, most of which occur in sub-Saharan Africa and Southeast Asia including China [13,14,15].

Pre-existing liver disease due to HBV or hepatitis C virus (HCV) infections may compromise the ability of hepatocytes to inactivate carcinogens such as aflatoxin [16]. We found a high prevalence of hepatitis infection in a study population in the Ejura Sekyedumase district, an Ashanti region of Ghana [17]. Forty-three of 140 participants (30.7%) tested positive for HBV or HCV infections; 23 (16.4%) for HBV and 20 (14.3%) for HCV; three participants were positive for both HBV and HCV infections. In another study that we conducted among HIV positive people in Kumasi, 11% were HBV positive and 1.3% HCV positive [18]. HBV infection was marginally associated with high aflatoxin biomarker levels and HBV positive participants had 2.38 times greater risk of having high aflatoxin B_1_ biomarker levels than HBV negative. The World Health Organization estimated that approximately 12% of the Ghanaian population has been infected with HBV and 2.8% with HCV [19]. In Kumasi the prevalence of HBV and HCV was previously reported to be 15% and 2.3% respectively [20].

This study was designed to determine aflatoxin biomarker levels in HBV and HCV positive patients compared with HBV and HCV negative controls and examine the association between aflatoxin biomarker levels and liver disease or liver cancer. A secondary objective was to examine predictors of high aflatoxin biomarker levels in study participants. As with most chronic diseases in Africa, patients with liver disease/cancer seek medical attention only when it is too late for any meaningful intervention to be implemented. Therefore, this study was conducted to provide data that can be used to formulate educational and other interventions to decrease aflatoxin intake, improve the quality of life, and reduce the high mortality associated with liver cancer in Ghana. In Ghana, the commonest cause of cancer death in females was malignancies of the breast (Age-Standardized Cancer Ratio, 17.24%), followed closely by hematopoietic organs (14.69%), liver (10.97%) and cervix (8.47%); whilst in males, the highest cancer mortality was from liver (21.15%), followed by prostate (17.35%), hematopoietic organs (15.57%), and stomach (7.26%) [21].

## 2. Experimental Section

### 2.1. Methods

A case-control study was conducted among patients attending clinics at the Komfo Anoyke Teaching Hospital (KATH) in Kumasi, the capital city of the Ashanti region of Ghana, from June 2012 to August 2013. KATH is a 1200-bed facility in the city of Kumasi and the second-largest hospital in Ghana [22]. It is the teaching hospital of the Kwame Nkrumah University of Science and Technology (KNUST) Medical School. KATH is the only tertiary health institution in the Ashanti Region and serves a population of 10 million people who primarily reside in Ashanti, Brong Ahafo, Northern, Upper East and Upper West Regions of Ghana [23]. Cases were defined as HBV/HCV positive patients who were clinically diagnosed with liver disease (e.g., alanine aminotransferase (ALT) level ≥60) or liver cancer that were biochemically confirmed through liver function tests and imaging with liver ultrasound and liver fibroscan. Two groups of controls were recruited for the study from the Transfusion Medicine Unit at KATH. Positive control participants were identified as patients diagnosed with HBV/HCV without any evident symptoms of acute liver damage and ALT test scores within the normal range. Negative controls were people who came in for routine blood donations, who tested negative for HBV and HCV, and who self-reported no liver problems. The response rate in the study was 80%.

Participants were Ghanaian men and women who were 18 years of age and older, and who belonged to the different ethnic groups representative of the Ashanti region, such as, the Akans, Gruma, Busanga, Dagbani, Basare, Moshi, Hausa, Ewe and Dagati. After informed consent was obtained, participants were asked to complete an interviewer-administered questionnaire that included questions on: (1) socio-demographic factors (age, sex, marital status, income, education, religion, and occupation); and (2) eating habits (basic staple foods, food acquisition, storage and preparation). A private room was used for interviewing to ensure confidentiality. A sample of freshly voided urine was collected to screen for the aflatoxin M_1_ metabolite. The participants’ medical records were reviewed and clinical data, such as liver function tests, liver ultrasounds, fibroscans, and stage of liver cancer, were abstracted.

### 2.2. Determination of Aflatoxin M_1_ Levels in Urine

Urine samples were centrifuged at 2300 rpm, and 5.0 mL of supernatant was collected, acidified with 0.5 mL of 1.0 M ammonium formate (pH 4.5) and diluted with water to a total volume of 10.0 mL. Samples were then loaded onto a 3 mL preparative Aflatest WB immunoaffinity column (VICAM, Watertown, MA, USA) at a flow rate of 1 mL/min. Following washing of the column the aflatoxin fraction was eluted from the column with 2 mL of 80% methanol, dried under N2 and re-suspended in 200 mL of a 1:1 solution of methanol:20 mM ammonium formate. Samples were analyzed using a Shimadzu HPLC system (Shimadzu Corporation, Kyoto, Japan) with fluorescence detection capabilities. A 250 4.6 mm LiCrospher RP-18 column with pore size 100 Å and particle size 5 mm (Alltech Associates, Deerfield, IL, USA) was used to resolve aflatoxin metabolites. The mobile phase consisted of 22% ethanol buffered with 20 mM ammonium formate (pH 3.0) in water. Isocratic elution of the mobile phase for 20 min at a rate of 1 mL/min allowed for proper chromatographic separation. External AFM_1_ standards were prepared weekly and injected following every five injections of samples. The limit of detection for this method was 0.5 pg/mg of urine for AFM_1_. Urinary AFM_1_ concentrations were expressed as pg/mL creatinine to correct for variations in urine dilution among samples. Creatinine concentrations were measured by a Selectra E auto-analyzer (Vital Scientific, Dieren, The Netherlands).

### 2.3. Statistical Analysis

Analysis was performed on 276 participants (38 cases, 136 negative controls and 102 positive controls). Bivariate analyses (chi-square and Fisher’s exact tests for categorical variables and *t*-test and analysis of variance for continuous variables) were used to compare differences between the three groups. Factors that had *p* ≤ 0.1 were carried forward to the multivariate model. This more conservative *p* ≤ 0.1 was selected in order to protect against residual confounding. Because there were three groups for the logistic model various comparisons were performed. In the first model cases were compared to both positive and negative controls, the second model included cases *versus* positive controls only, and the third was a comparison of cases and negative controls only. Logistic regression was used for all models to estimate odds ratios (ORs) and 95% confidence intervals (CIs) for the association between sociodemographic variables, aflatoxin awareness and food consumption practices, and being a case when compared to other groups. SAS 9.4 (SAS institute, Cary, NC, USA) was used for analyses. For all statistical tests a two-sided *p*-value < 0.05 was considered significant.

## 3. Results

### 3.1. Sociodemographic and Clinical Characteristics of the Study Groups

Participants differed according to sex and age (Table 1). A much higher percentage of participants in all groups were male. Cases were significantly older when compared to negative and positive controls (*p* = 0.0370). Most participants were Akans, Christians, employed, and had junior secondary school (JSS)/secondary education. Mean AFM_1_ levels (pg/mg creatinine) for cases (68.52 ± 679.8), positive controls (65.28 ± 334.1) and negative controls (67.02 ± 160.1) did not differ significantly from each other (Table 2). Although the median AFM_1_ levels was higher for cases (9.94 pg/mg) than for negative (7.72 pg/mg) and positive (6.76 pg/mg) controls the difference was also not significant. However, levels of the liver enzymes, aspartate transaminase (AST) and alanine transaminase (ALT) were significantly different between cases and positive controls. Mean levels of AST and ALT for positive controls remained within normal levels for these liver enzymes, while the mean levels of both AST and ALT for cases were above normal clinical ranges. AST and ALT tests were not done for negative controls due to limited resources. Liver disease was excluded based on HBV and HCV negative status and reports of no liver problems by these participants.

### 3.2. Food Preparation and Consumption Habits

There was statistically significant difference among the groups regarding who prepared the food they ate. Approximately 43% of positive controls reported preparing their food themselves compared to 32% of cases and 18.5% of negative controls. Approximately 48% of both cases and negative controls reported that their wife or another female family member prepared their food compared to 31% of positive controls (Table 3).

### 3.3. Aflatoxin Awareness and Knowledge of Study Participants

A slightly higher percent of cases than the other groups reported that they had heard of aflatoxin previously, but the difference was not significant (Table 4). A higher percentage of cases than either of the control groups also reported being aware of aflatoxin contamination of groundnuts and maize. However, most cases (82%) reported that aflatoxin contamination cannot cause sickness in humans. Awareness of aflatoxin contamination of crops and of the relationship of aflatoxin to disease was low for all three groups.

### 3.4. Sociodemographic Factors, Consumption Habits and Awareness of Aflatoxin as Predictors of Being a Case

Multivariable analysis showed that age was a significant predictor for being a case when cases were compared to the negative controls (Table 5). The odds of being a case was 70% less for participants aged 25–34 years (OR 0.30, 95% CI 0.10–0.88). Akans were seven times more likely to have AFM_1_ levels below the median for cases when compared to other ethnic groups, which include Gruma, Busanga, Dagbani, Basare, Moshi, Hausa, Ewe and Dagati (OR 7, CI 1.41–34.68). In the model in which cases were compared to positive controls, cases were 2.29 times more likely to report awareness of aflatoxin contamination of groundnuts (95% CI 1.06–4.91) and 2.5 times more likely to report awareness of aflatoxin contamination of maize (95% CI 1.17–5.39). Cases were also two times more likely to report awareness of aflatoxin contamination of maize than all controls combined (95% CI 17–5.39) (Table 5).

## 4. Discussion

### 4.1. Sociodemographic and Clinical Characteristics of the Study Groups

It is interesting to note that a much higher percentage of participants in all groups were male. It is known that men are twice as likely to develop liver cancer compared to women, and that in developing countries the rate of liver cancer for men is 10 times that of women. It is believed that males produce higher levels of interleukin-6 (IL-6), that promotes inflammation in response to liver injury, than females, and that production of IL-6 is suppressed by estrogen [24]. IL-6 contributes to the chronic liver inflammation that leads to cancer. This difference in males and females has been found to be due to different upregulation or deregulation of genes associated with inflammatory pathways. Females were found to be less vulnerable to “gender specific genes” than males [25]. When comparing the age of the participants, cases had a statistically significant higher average age than either of the control groups. This is not unexpected as it takes two or more decades for development of hepatocellular carcinoma [26].

Our finding of no significant difference in mean AFM_1_ levels among cases, positive controls and negative controls may be a result of the constant daily exposure of all three groups to aflatoxin in the diet. The higher median AFM_1_ levels among cases may be the result of greater aflatoxin intake or as a result of liver damage. The AFM_1_ metabolite is excreted in urine in 24–48 h after aflatoxin ingestion [27]. Although we realize that a single AFM_1_ measurement is not the ideal way to examine the association between aflatoxin exposure and liver disease, we were unable to do multiple AFM_1_ measurements or to determine the AFB_1_ biomarker, which indicates more long-term aflatoxin exposure (2–3 months or more). However, because of the chronic dietary exposure of people in this region of Ghana to aflatoxin, urinary excretion of AFM_1_ can almost always be detected reliably. In a previous study that we conducted in Ghana, we found AFM_1_ in at least 91% of study participants [28]. We also found AF-ALB in 100% of these participants indicating concurrent presence of both biomarkers [28].

As expected, mean levels of liver enzymes, AST and ALT, were significantly different between cases and positive controls. Mean levels of AST and ALT were above normal clinical ranges for cases but remained within normal levels for positive controls.

Akans were seven times more likely to have AFM_1_ levels below the median aflatoxin level for cases when compared to those in other ethnic groups (OR 7, CI 1.41–34.68). The diet of Akans seems to be more varied in that they eat other products, such as, yams, rice and plantains compared to ethnic groups in the society (the Gruma, Busanga, Dagbani, Basare, Moshi, Hausa, Ewe and Dagati) who may eat mainly groundnut, maize and other cereal-based diets. We found previously in a study conducted in Ghana that ethnicity tended to be a predictor of high AFB_1_ levels. Participants belonging to the Dagbani, Basare/Basali, Gonja and Konkomba ethnic group tended to be 2.8 times as likely to have high AFB_1_ levels compared to those from the Akan group [28].

Awareness of aflatoxin contamination of crops and of the relationship of aflatoxin to disease was low among all three groups. However, in the model in which cases were compared to positive controls, cases were 2.29 times more likely to report awareness of aflatoxin contamination of groundnuts (95% CI 1.06–4.91), and 2.52 times more likely to report aflatoxin contamination of maize (95% CI 17–5.39) (Table 5). Regrettably, most of these cases reported that aflatoxin contamination does not cause sickness in humans. This shows that awareness of aflatoxin contamination may occur without proper understanding of the serious potential adverse health impacts of the toxin. Another study conducted in Ghana reported the difficulty in guaranteeing the receptiveness of public awareness campaigns should they contradict beliefs, particularly because maize-based foods are staple to the average Ghanaian diet [29]. As a result there is continued consumption of aflatoxin-contaminated foods despite aflatoxin awareness.

### 4.2. Food Preparation and Consumption Habits

A statistically significant higher percent (43%) of positive controls reported preparing their own food compared with cases (32%) and negative controls (18.5%). Approximately 48% of both cases and negative controls reported that their wife or another female family member prepared their food compared to 31% of positive controls. Spouses and female family members may not be careful to select wholesome grains, to store grains properly, and to sort out contaminated grains before cooking. This could have placed cases at high risk for aflatoxin exposure over time. Negative controls are at this same risk but are younger and so do not yet show the effect of chronic aflatoxin exposure. A study conducted in Ghana to assess the relationship between food preparation, contamination awareness, and food safety found that although individuals responsible for preparing food, as well as consumers, were aware of possible food contamination, this knowledge was not reflective in their preparation or consumption practices [29]. A factor that should be considered when questioning the effectiveness of aflatoxin contamination awareness is the economic status of study participants since many participants expressed that their food preparation and consumption practices were heavily influenced by whether or not they could afford to discard seemingly unsafe foods. This was true for consumers as well as farmers and vendors.

### 4.3. Limitations

There are several limitations of this study that should be considered in interpreting the results. The first is the small number of cases that we were able to recruit which likely precluded us from obtaining some significant results. The age difference between cases and controls is a limitation that was difficult to overcome, partly because cancer develops after two or more decades, but also because we had to recruit controls from the Transfusion Medicine Unit of the hospital in order to know their HBV and HCV infection status. An additional limitation is the use of a single AFM_1_ measurement to examine the association between aflatoxin exposure and liver disease, but limited resources prevented us from conducting multiple AFM_1_ measurements.

## 5. Conclusions, Potential Interventions and Future Research

Despite the limitations, this study identifies factors that contribute to aflatoxin ingestion in this population, and points to interventions that, if implemented, may reduce aflatoxin exposure and possibly decrease liver disease and liver cancer in the population. The finding of high AFM_1_ levels in urine of all groups of study participants confirms chronic dietary exposure to aflatoxin. The significant finding of higher levels of awareness of aflatoxin contamination in maize and groundnuts among the cases without proper understanding of the serious potential adverse health impact of aflatoxin exposure, indicates the need for educational programs that address awareness of the harmful health effects of aflatoxin, emphasize that these harmful effects occur as a result of years of chronic exposure to the toxin, and stress that current attention to the problem is crucial. The preponderance of males in the study sample highlights the higher liver cancer risk for males and indicates that this increased risk for males should be emphasized in educational awareness and intervention programs. The significantly lower median AFM_1_ levels observed among Akans compared to the other ethnic groups in the study may indicate that the more varied diet observed in Akans [28] could result in less aflatoxin exposure. If further investigation into the diets of the different groups show that some groups eat mainly foods that are more prone to aflatoxin contamination, then the information could be used in developing interventions to assist groups in the society to vary their diets using available and inexpensive local food crops. This may prove to be effective in decreasing aflatoxin exposure. Interventions that incorporate simple pre- and post-harvest strategies to decrease fungal proliferation, and build-up of aflatoxin, in foods, and address simple food selection, storage, preparation, and consumption approaches to decrease aflatoxin exposure are also urgently needed. Further research concerning the effects of aflatoxin exposure levels in HBV and HCV positive and negative people are needed to advocate for effective regulatory policies to reduce aflatoxin contamination in food. These regulations may potentially decrease aflatoxin exposure levels in Ghana and other countries where staple crops are highly prone to aflatoxin contamination.

## Figures and Tables

**Table 1:** Sociodemographic characteristics of study participants.

Variable	Cases *n* = 38	Negative Controls *n* = 136	Positive Controls *n* = 102	*p*-Value
Sex	–	–	–	**<0.0001**
Male	29 (76.3%)	119 (88.1%)	66 (64.7%)	–
Female	9 (23.7%)	16 (11.8%)	36 (35.3%)	–
Age	–	–	–	**0.0370**
Median	39	32	31	–
Mean and SD	37.4 + 11.4	32 + 8.7	33.1 + 11	–
≤24	4 (10.5%)	27 (20%)	25 (24.5%)	–
25–34	11 (29%)	64 (47.4%)	36 (35.3%)	–
35–44	15 (39.5%)	30 (22.2%)	23 (22.6%)	–
≥45	8 (21.1%)	14 (10.4%)	18 (17.7%)	–
Education	–	–	–	0.1813
None	4 (16.7%)	5 (5.1%)	5 (6.7%)	–
Primary	2 (8.3%)	4 (4.1%)	2 (2.7%)	–
JSS/Secondary/Form/Technical	14 (58.3%)	68 (69.4%)	44 (58.7%)	–
College/University	4 (16.7%)	21 (21.4%)	24 (32%)	–
Ethnicity	–	–	–	0.1223
Akan	28 (73.7%)	96 (71.1%)	84 (82.3%)	–
Other	10 (26.3%)	40 (28.9%)	18 (17.7%)	–
Religion	–	–	–	0.0937
Christian	32 (84.2%)	112 (82.9%)	94 (92.2%)	–
Muslim/Other	6 (15.8%)	23 (17.1%)	8 (7.8%)	–
Employment	–	–	–	0.0625
Yes	31 (81.6%)	108 (80%)	69 (67.6%)	–
No	7 (18.4%)	27 (20%)	33 (32.4%)	–
Total household size	–	–	–	0.8013
0–9	32 (84.2%)	109 (80.7%)	88 (86.3%)	–
10–19	4 (10.5%)	20 (14.8%)	10 (9.8%)	–
20 and above	2 (5.3%)	6 (4.4%)	4 (3.9%)	–

JSS = Junior Secondary School. Other ethnic groups include Gruma, Busanga, Dagbani, Basare, Moshi, Hausa, Ewe and Dagati.

**Table 2:** Aflatoxin M_1_, aspartate transaminase (AST) and alanine transaminase (ALT) levels for the study groups.

AFM_1_, AST and ALT	Cases *n* = 38	Negative Controls *n* = 136	Positive Controls *n* = 102	*p*-Value
**AFM_1_** (pg/mg creatinine)	–	–	–	–
Mean ± SD	68.52 ± 679.8	67.02 ± 160.1	65.28 ± 334.1	0.9463
Median and Range	9.94; 0–679.88	7.72; 0–1173.09	6.76; 0–3019.69	–
**AST** (U/L)	*n* = 16	Not done	*n* = 11	–
Mean ± SD	184.74 ± 276.7	–	24.86 ± 5.2	**0.0355**
Median and Range	83.55; 18.7–1053.2	–	26; 15–32	–
**ALT** (U/L)	*n* = 21	Not done	*n* = 32	–
Mean ± SD	88 ± 53.7	–	27.25 ± 13.4	**<0.0001**
Median and Range	90; 11–199	–	22; 11–66	–

**Table 3:** Food preparation and consumption habits of participants.

Variable	Cases *n* = 38	Negative Controls *n* = 136	Positive Controls *n* = 102	*p*-Values
**Who prepares your food?**	–	–	–	**0.0009**
Self	12 (31.6%)	25 (18.5%)	44 (43.1%)	–
Wife or other female family member	18 (47.4%)	66 (48.9%)	32 (31.4%)	–
Variation	8 (21%)	44 (32.6%)	26 (25.5%)	–
**Groundnut frequency**	–	–	–	0.4887
Never	21 (55%)	62 (45.6%)	53 (52%)	–
1–3 times	17 (45%)	73 (53.7%)	49 (48%)	–
Everyday	0 (0%)	1 (0.7%)	0 (0%)	–
**Maize frequency (on the cob)**	–	–	–	0.2816
Never	11 (29%)	33 (24.3%)	28 (27%)	–
1–3 times	27 (71%)	102 (75%)	69 (68%)	–
Everyday	0 (0%)	1 (0.7%)	2 (2%)	–
**Groundnut paste or butter frequency**	–	–	–	0.4927
1 time or less per week	34 (94.4%)	118 (90.8%)	90 (96.8%)	–
2–3 times per week	2 (5.6%)	10 (7.7%)	3 (3.2%)	–
Everyday	0 (0%)	2 (1.5%)	0 (0%)	–
**Groundnut soup and sauce frequency**	–	–	–	0.4530
1 time or less per week	20 (60.6%)	64 (48.1%)	50 (53.8%)	–
2–3 times per week	13 (39.4%)	63 (47.4%)	37 (39.8%)	–
Everyday	0 (0%)	6 (4.5%)	6 (6.4%)	–
**Kenkey frequency**	–	–	–	0.4192
1 time or less per week	21 (58.3%)	59 (44.4%)	51 (52%)	–
2–3 times per week	14 (38.9%)	63 (47.4%)	39 (39.8%)	–
Everyday	1 (2.8%)	11 (8.3%)	8 (8.2%)	–
**Banku frequency**	–	–	–	0.1152
1 time or less per week	15 (42.9%)	42 (31.3%)	42 (43.7%)	–
2–3 times per week	19 (54.3%)	75 (56%)	43 (44.8%)	–
Everyday	1 (2.8%)	17 (12.7%)	11 (11.5%)	–
**Apeprensa/Asana frequency**	–	–	–	0.3399
1 time or less per week	38 (100%)	132 (98.5%)	96 (97%)	–
2–3 times per week	0 (0%)	2 (1.5%)	3 (3%)	–
Everyday	0 (0%)	0 (0%)	0 (0%)	–

Numbers for variables may not always add up to the total number due to missing responses.

**Table 4:** Aflatoxin awareness and knowledge among study participants.

Variable	Cases *n* = 38	Negative Controls *n* = 136	Positive Controls *n* = 102	*p*-Value
Have you heard of aflatoxin before?				0.1479
Yes	22 (57.9%)	65 (48.5%)	41 (40.2%)	–
No	16 (42.1%)	69 (51.5%)	61 (59.8%)	–
Are you aware of aflatoxin contamination of groundnuts?				0.0547
Yes	19 (50%)	57 (42.5%)	31 (30.4%)	–
No	19 (50%)	77 (57.5%)	71 (69.6%)	–
Are you aware of aflatoxin contamination of maize?				0.0506
Yes	22 (57.9%)	59 (44%)	36 (35.3%)	–
No	16 (42.1%)	75 (56%)	66 (64.7%)	–
Can aflatoxin cause sickness in humans?				0.0886
Yes	7 (18.4%)	39 (29.1%)	18 (17.6%)	–
No	31 (81.6%)	95 (70.9%)	84 (82.4%)	–

**Table 5:** Logistic Models of probability of being a case given food consumption habits and awareness of aflatoxin.

Variable	Case *vs.* All Controls *	Cases *vs.* Negative Controls *	Case *vs.* Positive Controls *
**Age**	–	–	–
≤24	0.30 (0.08–1.10)	0.25 (0.06–1.01)	0.36 (0.09–1.38)
25–34	0.44 (0.16–1.18)	**0.30 (0.10–0.88)**	0.68 (0.23–2.01)
35–44	1.13 (0.43–2.96)	0.87 (0.30–2.54)	1.46 (0.51–4.22)
≥45	Referent	Referent	Referent
**Who prepares your food**	–	–	–
Self	Referent	Referent	Referent
Wife or other female family member	1.06 (0.48–2.33)	0.56 (0.24–1.34)	2.06 (0.87–4.87)
Variation	0.66 (0.25–1.71)	0.37 (0.13–1.05)	1.12 (0.40–3.12)
**Maize consumption frequency**	–	–	–
Never	Referent	Referent	Referent
1–3 times	0.43 (0.16–1.17)	0.47 (0.17–1.32)	0.39 (0.14–1.12)
Everyday	0.95 (0.20–4.52)	0.75 (0.15–3.76)	1.48 (0.23–9.33)
**Banku consumption frequency**	–	–	–
1 time or less per week	Referent	Referent	Referent
2–3 times per week	0.90 (0.43–1.87)	0.71 (0.32–1.54)	1.23 (0.55–2.75)
Everyday	0.20 (0.02–1.58)	0.16 (0.02–1.34)	0.25 (0.03–2.14)
**Awareness of groundnut contamination**	–	–	–
Yes	1.69 (0.85–3.37)	1.36 (0.66–2.18)	**2.29 (1.06–4.91)**
No	Referent	Referent	Referent
**Awareness of maize contamination **	–	–	–
Yes	**2.05 (1.02–4.11)**	1.77 (0.85–3.66)	**2.52 (1.17–5.39)**
No	Referent	Referent	Referent
**Knowledge of causing disease in humans**	–	–	–
Yes	0.71 (0.29–1.71)	0.55 (0.22–1.36)	1.05 (0.40–2.76)
No	Referent	Referent	Referent

* Models adjusted for gender, education, ethnicity, employment status and religion.
